# Inflammatory linear verrucous epidermal nevus successfully treated with ustekinumab

**DOI:** 10.1016/j.jdcr.2024.12.031

**Published:** 2025-01-15

**Authors:** Sara Almarzooqi, Shaden Abdelhadi

**Affiliations:** Dermatology Department, Sheikh Khalifa Medical City, Abu Dhabi, United Arab Emirates

**Keywords:** inflammatory linear verrucous epidermal nevus, ustekinumab

## Introduction

Inflammatory linear verrucous epidermal nevus (ILVEN) is a notoriously difficult condition to treat. In fact, since it was initially characterized in 1971,^1^ resistance to traditional psoriasis treatment has remained a diagnostic criterion. A recent publication has revealed a mosaic variant in the *CARD14* gene in 2 patients with ILVEN,^2^ resulting in successful treatment with ustekinumab. We highlight a case successfully treated at our facility, adopting a similar approach.

## Case presentation

A 13-year-old male presented to our clinic for evaluation of a pruritic skin lesion on his flank. The lesion first developed at the age of 2 years and has slowly grown, only to stabilize in size 1 year prior to presentation. He was also known to have mild atopic dermatitis controlled with emollients and asthma. A review of systems, past surgical, and family history were noncontributory.

**Fig 1:**
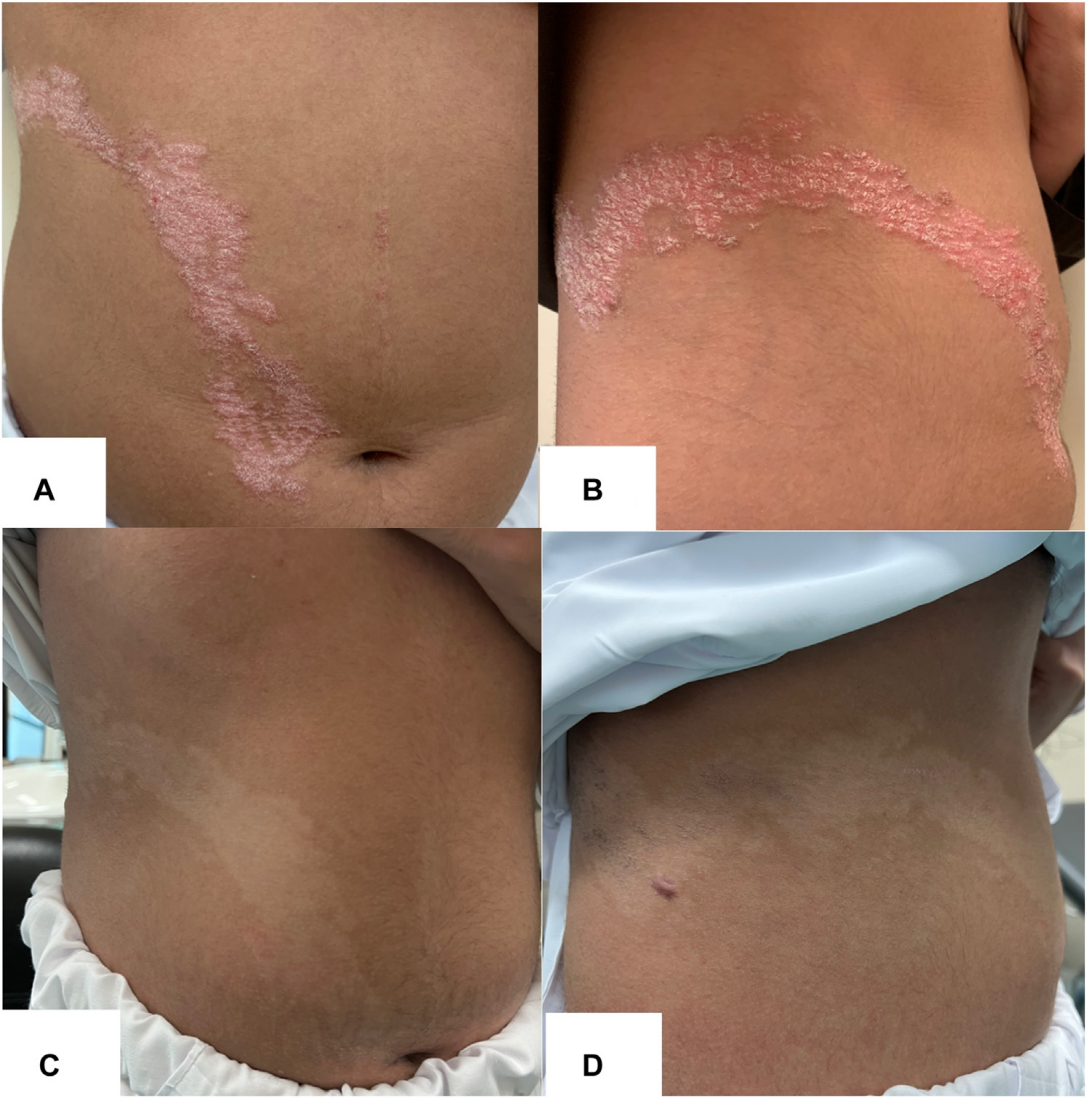
**A** and **B,** Before starting treatment. **C** and **D,** Six months after treatment with ustekinumab.

**Fig 2:**
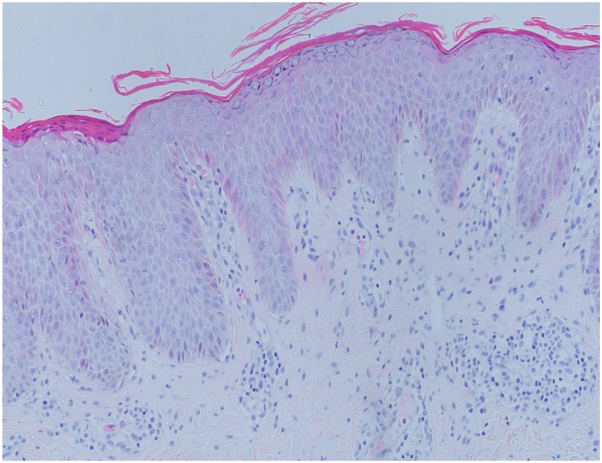
Hematoxylin and eosin demonstrating parakeratosis with underlying hypogranulosis alternating with orthokeratosis with underlying hypergranulosis, and a perivascular lymphohistiocytic infiltrate in the upper dermis.

On examination, a well-demarcated erythematous plaque with overlying thick white scales was noted in a linear blaschkoid distribution overlying the right abdomen, flank, and back. Multiple discrete papules were also present on the midline of the abdomen (Fig 1, *A* and *B*). The nails, scalp, mucous membranes, and musculoskeletal system were unaffected. A biopsy revealed parakeratosis with underlying hypogranulosis alternating with orthokeratosis with underlying hypergranulosis and a perivascular lymphohistiocytic infiltrate in the upper dermis (Fig 2). No evidence of porokeratosis was identified on histopathological examination. A clinicopathologic diagnosis of ILVEN was established while also keeping linear psoriasis, a closely related entity, in the differential diagnosis. Genetic testing performed on lesional skin biopsy revealed no clinically relevant variants, including in the *CARD14* gene.

The patient was initially started on calcipotriol/betamethasone dipropionate gel along with emollients for 3 months, providing a partial improvement. He was later switched to an alternate regimen of morning tacrolimus and evening tretinoin to be used 5 days a week with calcipotriol/betamethasone dipropionate gel on weekends. The response to topical treatments had fluctuated and correlated with the patient’s compliance. The decision to begin ustekinumab was taken 9 months later as the patient did not achieve satisfactory results. Ustekinumab treatment was initiated using a classic psoriasis dosing of 45 mg at week 0, week 4, and every 12 weeks after that. After only 2 injections, a remarkable response was obtained, and almost complete clearance was achieved after 6 months (Fig 1, *C* and *D*). The patient still receives the maintenance dose with no reported side effects, and his routine monitoring laboratory investigations remain unremarkable.

## Discussion

Recalcitrance to treatment is one of the criteria used to diagnose ILVEN,^1^ along with early age of onset, an intensely pruritic linear verrucous plaque distributed along the lines of Blaschko, and an inflammatory and psoriasiform histologic appearance. The differential diagnosis includes linear psoriasis, linear porokeratosis, linear lichen planus, lichen striatus, verrucous epidermal nevus, and CHILD nevus. Whether ILVEN and linear psoriasis represent the same entity is still debatable. Factors favoring separating the 2 include differences in clinical course, absence of family history, poor response to traditional psoriasis treatments, and differences in immunohistochemistry studies. The latter include the absence of involucrin in the parakeratotic areas of ILVEN,^3^ lower Ki-67+ nuclei,^4^ and higher Keratin-10+ cells, and HLA-DR expression as compared to psoriasis.^4^ Additionally, the density of CD8+, CD45RO+, CD2+, CD94, and CD161 was found to be markedly reduced in ILVEN versus psoriasis.^4^ Rarely, the 2 conditions may also coexist.

A variety of therapeutic modalities have been described in literature, including topicals (eg, corticosteroids, calcipotriol, 5-fluorouracil, crisaborole), oral acitretin, cryotherapy, and lasers (eg, CO2, Excimer 308 nm). A combination of these treatments and a long-term course are often required. To date, the only curative treatment is surgical excision. With the advent of biological therapies, attempts to treat with etanercept,^5^ ustekinumab,^2^ and most recently brodalumab^6^ have been published.

In one publication, 2 ILVEN patients were identified to have heterozygous missense gain-of-function somatic mutation in the Caspase Recruitment Domain Protein 14 (*CARD14*) gene,^2^ which leads to elevated levels of IL-12 and IL-23 via NF-κB- and non-NF-κB-dependent manners, explaining ustekinumab’s effectiveness as a treatment option. However, our patient responded well to treatment with ustekinumab despite not having the *CARD14* gene variant. This suggests that the mechanism of action of ustekinumab in ILVEN might not be exclusively related to the *CARD14* gene, and therefore, patients without this variant may also benefit from treatment. Interestingly, one of those *CARD14* variants was previously reported to cause pityriasis rubra pilaris (PRP) when present as a germline mutation.^2^^,^^7^ Furthermore, a germline variant of the Gap Junction Protein Alpha 1 (*GJA11*) gene previously described in erythrokeratodermia variabilis et progressiva (EKVP)^8^ was discovered to cause ILVEN when present in a mosaic pattern.^8^^,^^9^ In another case report, a patient diagnosed with ILVEN in childhood and later developed psoriasis vulgaris elsewhere was found to have a germline *CARD14* pathogenic variant.^10^ Such findings imply that ILVEN may result in more widespread disease in the offspring; if mosaic variants were also present in the gonads (gonosomal mosaicism), they can hypothetically be passed on as germline variants. Therefore, effective counseling is vital for such patients. In addition, genetic analysis in a patient initially diagnosed with ILVEN revealed a mutation in the phosphomevalonate kinase (PMVK) gene, rendering the diagnosis linear porokeratosis and allowing successful treatment with lovastatin/cholesterol cream.^10^ Two other patients with ILVEN were found to have mutations in the NAD(P)H steroid dehydrogenase-like protein (NSDHL) gene, changing the diagnosis to CHILD nevus.^10^ This emphasizes the importance of genetic testing in patients with ILVEN for appropriate management.

While genetic research continues to reshape our understanding of this condition and provides hope for future tailored therapies, realistic treatment outcomes should be established with patients and parents in the meantime. Additionally, while ustekinumab appears to be effective, further investigation is still required to confirm its role.

## Conflicts of interest

None disclosed.

## References

[1] Altman J., Mehregan A.H. (1971). Inflammatory linear verrucose epidermal nevus. Arch Dermatol.

[2] Riachi M., Polubothu S., Stadnik P. (2021). Molecular genetic dissection of inflammatory linear verrucous epidermal naevus leads to successful targeted therapy. J Invest Dermatol.

[3] Ito M., Shimizu N., Fujiwara H., Maruyama T., Tezuka M. (1991). Histopathogenesis of inflammatory linear verrucose epidermal naevus: histochemistry, immunohistochemistry and ultrastructure. Arch Dermatol Res.

[4] Vissers W.H., Muys L., Erp P.E., de Jong E.M., van de Kerkhof P.C. (2004). Immunohistochemical differentiation between inflammatory linear verrucous epidermal nevus (ILVEN) and psoriasis. Eur J Dermatol.

[5] Renner R., Colsman A., Sticherling M. (2008). ILVEN: is it psoriasis? Debate based on successful treatment with etanercept. Acta Derm Venereol.

[6] Green R., Hunter C., Chia J.C. (2022). A case of inflammatory linear verrucous epidermal nevus successfully treated with Brodalumab. Pediatr Dermatol.

[7] Lwin S.M., Hsu C.K., Liu L., Huang H.Y., Levell N.J., McGrath J.A. (2018). Beneficial effect of ustekinumab in familial pityriasis rubra pilaris with a new missense mutation in CARD14. Br J Dermatol.

[8] Umegaki-Arao N., Sasaki T., Fujita H. (2017). Inflammatory Linear Verrucous Epidermal Nevus with a Postzygotic GJA1 Mutation Is a Mosaic Erythrokeratodermia Variabilis et Progressiva. J Invest Dermatol.

[9] Boyden L.M., Craiglow B.G., Zhou J. (2015). Dominant De Novo Mutations in GJA1 Cause Erythrokeratodermia Variabilis et Progressiva, without Features of Oculodentodigital Dysplasia. J Invest Dermatol.

[10] Atzmony L., Ugwu N., Hamilton C. (2022). Inflammatory linear verrucous epidermal nevus (ILVEN) encompasses a spectrum of inflammatory mosaic disorders. Pediatr Dermatol.

